# Diagnostic performance of chest CT in screening patients with suspected COVID-19 infection in a Western population

**DOI:** 10.1259/bjr.20200643

**Published:** 2020-07-29

**Authors:** Jasenko Krdzalic, Tom M.H. de Jaegere, Robert M. Kwee

**Affiliations:** 1 Department of Radiology, Zuyderland Medical Center, Heerlen/Sittard/Geleen, The Netherlands

## Abstract

**Objective:**

To investigate the diagnostic performance of chest CT in screening patients suspected of Coronavirus disease 2019 (COVID-19) in a Western population.

**Methods:**

Consecutive patients who underwent chest CT because of clinical suspicion of COVID-19 were included. CT scans were prospectively evaluated by frontline general radiologists who were on duty at the time when the CT scan was performed and retrospectively assessed by a chest radiologist in an independent and blinded manner. Real-time reverse transcriptase–polymerase chain reaction was used as reference standard. Sensitivity, specificity, positive predictive value (PPV), and negative predictive value (NPV) were calculated. Sensitivity and specificity of the frontline general radiologists were compared to those of the chest radiologist using the McNemar test.

**Results:**

56 patients were included. Sensitivity, specificity, PPV, and NPV for the frontline general radiologists were 89.3% [95% confidence interval (CI): 71.8%, 97.7%], 32.1% (95% CI: 15.9%, 52.4%), 56.8% (95% CI: 41.0%, 71.7%), and 75.0% (95% CI: 42.8%, 94.5%), respectively. Sensitivity, specificity, PPV, and NPV for the chest radiologist were 89.3% (95% CI: 71.8%, 97.7%), 75.0% (95% CI: 55.1%, 89.3%), 78.1% (95% CI: 60.0%, 90.7%), and 87.5% (95% CI: 67.6%, 97.3%), respectively. Sensitivity was not significantly different (*p* = 1.000), but specificity was significantly higher for the chest radiologist (*p* = 0.001).

**Conclusion:**

Chest CT interpreted by frontline general radiologists achieves insufficient screening performance. Although specificity of a chest radiologist appears to be significantly higher, sensitivity did not improve. A negative chest CT result does not exclude COVID-19.

**Advances in knowledge:**

Our study shows that chest CT interpreted by frontline general radiologists achieves insufficient diagnostic performance to use it as an independent screening tool for COVID-19. Although specificity of a chest radiologist appears to be significantly higher, sensitivity is still insufficiently high.

## Introduction

Coronavirus disease 2019 (COVID-19) has spread throughout the world and caused a pandemic.^1–6^ The social, health care and economic consequences of the COVID-19 pandemic are immense.^5^ Health-care systems throughout the world have been overloaded.^7^ It is important to protect the often vulnerable hospitalized patients and health-care workers from becoming infected with COVID-19. Patients with suspected COVID-19 should be rapidly triaged and isolated.^8^ Real-time reverse-transcriptase–polymerase chain reaction (RT-PCR) is considered the gold-standard to diagnose COVID-19.^9–13^ However, RT-PCR testing is time-consuming,^14,15^ which puts pressure on the isolation room capacity in hospitals. In addition, an initial negative RT-PCR result does not entirely rule out COVID-19. If clinical suspicion persists, patients should be retested.^16,17^Another limitation is that there is currently a relative lack of RT-PCR testing capacity.^3^ Ai et al^18^ suggested that chest CT could be used as a rapid screening tool in patients with suspected COVID-19. Their study, which included more than 1000 patients from China, reported that the sensitivity of chest CT for the diagnosis of COVID-19 was 97%.^18^ However, other smaller studies reported that chest CT can miss a considerable number of COVID-19 patients: In Bernheim et al.'s study,^19^ 20/36 (56%) of COVID-19 patients with symptoms up to 2 days before chest CT had negative CT findings. In Pan et al.'s study,^20^ initial negative CT findings were present in 4/21 (19%) of COVID-19 patients. In Yang et al.'s study,^21^ there were 17/149 (11%) symptomatic COVID-19 patients with negative CT findings. As such, it is not completely clear yet whether chest CT can be used as a reliable, independent screening tool. Furthermore, it is not clear either whether results from a Chinese population can be generalized to a Western population. Chest CT scans may be read by general radiologists or by dedicated chest radiologists. By using a pool of general radiologists, the hospital workload can be more evenly distributed. However, it is not clear yet whether general radiologists achieve similar diagnostic performance as a dedicated chest radiologist. Therefore, the purpose of our study was to investigate the diagnostic performance of chest CT in screening patients suspected of COVID-19 in a Western population.

## Methods and materials

The study followed the Standards for Reporting of Diagnostic Accuracy Studies (STARD) criteria.^22^

### Patients and CT protocol

This retrospective study was approved by the institutional review board of our hospital and patients’ consents were waived. Consecutive patients who underwent chest CT because of clinical suspicion of COVID-19 (*i.e.* presenting with fever, cough, and/or shortness of breath) in Zuyderland Medical Center, Heerlen/Sittard/Geleen, The Netherlands, between March 12, 2020 and March 20, 2020, were potentially eligible for inclusion. Patients with known COVID-19 (proven by RT-PCR testing) before CT scanning, were excluded. Cases who did not comply with the reference standard (see below) were also excluded. All patients underwent unenhanced chest CT on either a 64-slice CT scanner (Philips Incisive) or on a 64-slice dual source scanner (Siemens Somatom Definition Flash). Scanning parameters for both CT scanners were: collimation 64 × 0.625 or 0.6 mm,120 kVp, 667 max mA or 404 max mA, pitch1.0 or 1.2, and matrix size 512 × 512. CT images were reconstructed in the transverse plane with 1.0 mm slice thickness and 1.0 mm increment. Images were also reconstructed in axial, coronal, and sagittal planes with 3.0 mm slice thickness.

### CT analysis

#### Initial reading

**Figure 1.**
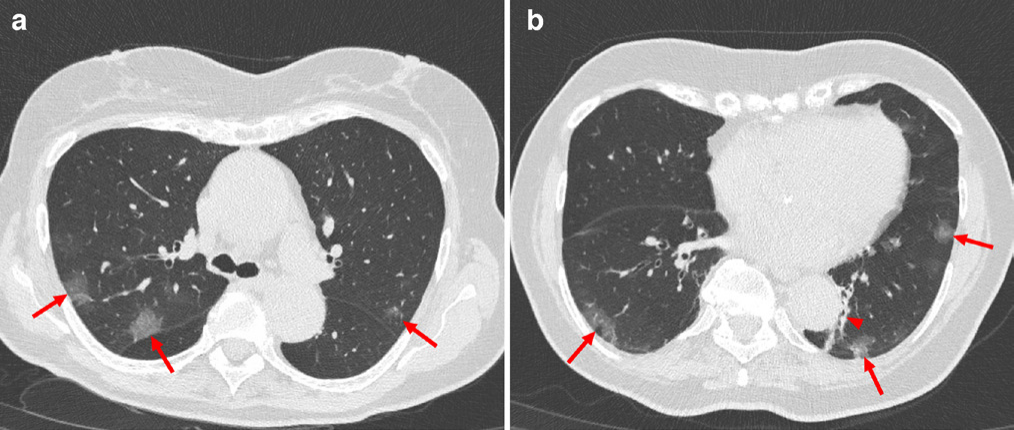
Typical CT features of peripherally distributed multifocal ground-glass opacities (red arrows) with posterior part (A, B)/lower lobe (B) pre-dilection in a 81-year-old female patient with COVID-19 who presented with presented with dyspnea and fever. Note; presence of atelectatic changes lateral from the descending aorta (arrowhead in B) and presence of minor respiration artifacts in the left lower lobe.

CT scans were initially read and reported by radiologists (*n* = 15) who were on duty at the time the CT scan was performed, as part of clinical care. These radiologists, who are referred to as the frontline general radiologists in the remainder of this scientific communication, all have experience in chest CT interpretation in the emergency setting during on-call hours (*e.g.* CT for suspected pulmonary embolism or trauma). The frontline general radiologists were aware of (some of the) CT features of COVID-19 pneumonia^23^ (Figure 1). However, at the time of reporting, there were no explicit threshold criteria published in the literature. Therefore, the final judgment was at the discretion of the frontline general radiologist. All reports of the frontline general radiologists were reviewed in consensus by two radiologists (*initials blinded for review*) and scored as follows: negative for (possible) COVID-19, positive for (possible) COVID-19, or equivocal. Equivocal cases were considered positive for (possible) COVID-19 in further analyses.

#### Retrospective reading

CT scans were retrospectively read by a chest radiologist (J.K. *initials blinded for review*) with 5 years of experience in chest CT interpretation who was aware of the clinical information as provided by the referring physician but blinded to the findings of the radiologists who made the initial report. The chest radiologist assessed the likelihood of COVID-19 using the COVID-19 Reporting and Data System (CO-RADS).^24^ CO-RADS uses a 5-point scale of suspicion for pulmonary involvement of COVID-19 on chest CT (CO-RADS 5: very high level of suspicion; CO-RADS 4: high level of suspicion; CO-RADS 3: equivocal findings, CO-RADS 2: low level of suspicion; and CO-RADS 1: very low level of suspicion).^24^ CO-RADS scores of 1–2 were considered negative and CO-RADS scores of 3–5 were considered positive for (possible) COVID-19.

#### Reference standard

Nasal and pharyngeal swab specimens were obtained for RT-PCR testing, according to WHO recommendation.^17^ Patients with negative initial RT-PCR result and persistent clinical suspicion (results of the first RT-PCR were available after 4 h) were retested. A patient with a positive RT-PCR result was considered to be infected with COVID-19, whereas a patient with (persistent) negative RT-PCR test result(s) was considered not infected with COVID-19.

#### Statistical analysis

Receiver operating characteristic (ROC) analysis was done using the likelihood scores of the chest radiologist, and area under the ROC curve (AUC) was calculated. Sensitivity, specificity, negative predictive value (NPV), and positive predictive value (PPV) of chest CT for the diagnosis of COVID-19 were calculated, along with 95% confidence intervals (CIs), both for the frontline general radiologists and for the chest radiologist who retrospectively read the CT scans. Sensitivity and specificity of the frontline general radiologists were compared to those of the chest radiologist by using the McNemar test. Statistical analyses were executed using MedCalc statistical software v. 12.6.0 (MedCalc Software, Ostend, Belgium).

## Results

**Figure 2.**
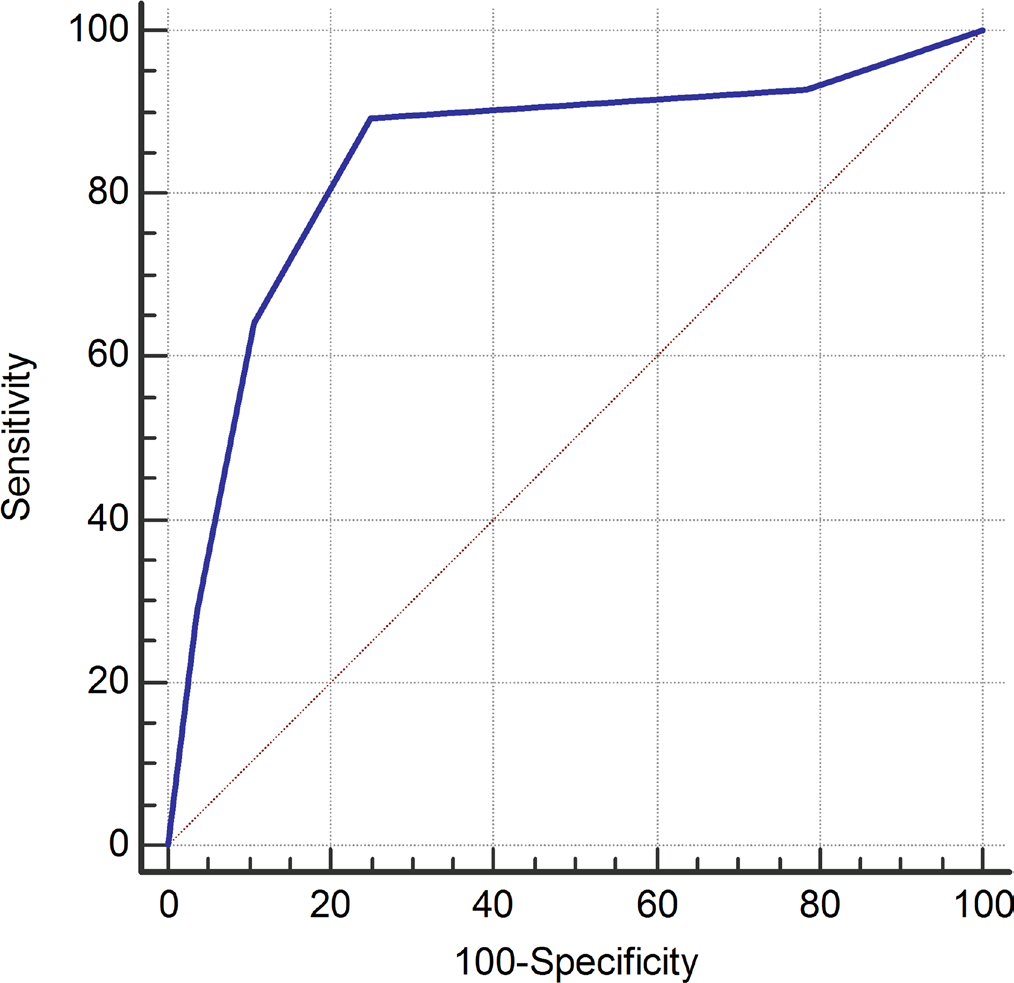
ROC curve for the diagnostic performance of the chest radiologist. AUC was 0.842. AUC, area under the curve; ROC, receiver operating characteristic.

60 consecutive patients (63.3% males, mean age 65.3 years [range 29–94]) were potentially eligible for inclusion. Four cases were excluded because they did not comply with the reference standard (three patients did not undergo initial RT-PCR testing, whereas one patient with negative initial RT-PCR result and persistent clinical suspicion did not undergo repeated RT-PCR testing). Time interval between chest CT and RT-PCR testing ranged between 0 and 4 days (median of 0 days). Based on the reference standard, 28 of the remaining 56 patients (50%) were infected with COVID-19. Duration of symptoms before chest CT was reported in 18 of 28 patients (64.3%) with COVID-19 (Table 1), with a median of 7 days (range 2–21 days). Diagnostic values of both the frontline general radiologists and the chest radiologist are displayed in Table 2. Sensitivity, specificity, PPV, and NPV for the frontline general radiologists were 89.3% (95% CI: 71.8%, 97.7%), 32.1% (95% CI: 15.9%, 52.4%), 56.8% (95% CI: 41.0%, 71.7%), and 75.0% (95% CI: 42.8%, 94.5%), respectively. Sensitivity, specificity, PPV, and NPV for the chest radiologist were 89.3% (95% CI: 71.8%, 97.7%), 75.0% (95% CI: 55.1%, 89.3%), 78.1% (95% CI: 60.0%, 90.7%), and 87.5% (95% CI: 67.6%, 97.3%), respectively. Number of false negative chest CT findings by duration of symptoms is displayed in Table 1. The ROC curve for the diagnostic performance of the chest radiologist is shown in Figure 2. AUC was 0.842. Sensitivity was not significantly different between the frontline general radiologists and the chest radiologist (*p* = 1.000). Specificity was significantly higher for the chest radiologist (*p* = 0.001).

**Table 1.** Duration of symptoms before chest CT and number of false negative chest CT findings in patients with COVID-19

Duration of symptoms	Number of patients	Number of false negative chest CT findings (frontline general radiologists)	Number of false negative chest CT findings (chest radiologist)
Early (0–2 days)	(*n* = 2)	1	1
Intermediate (3–5 days)	(*n* = 3)	1	0
Late (>6 days)	(*n* = 13)	0	1

**Table 2.** Diagnostic values of chest CT in screening patients with suspected COVID-19

Interpreters of chest CT	Sensitivity (95% CI)	Specificity (95% CI)	Positive predictive value (95% CI)	Negative predictive value (95% CI)
Frontline general radiologists (*n* = 15)	89.3% (71.8, 97.7)	32.1% (15.9, 52.4)	56.8% (41.0, 71.7)	75.0% (42.8, 94.5)
Chest radiologist	89.3% (71.8, 97.7)	75.0% (55.1, 89.3)	78.1% (60.0, 90.7)	87.5% (67.6, 97.3)

## Discussion

COVID-19 is a highly contagious disease and disseminates easily.^25^ Hospitals need to ensure that all infected patients are placed in strict isolation in order to prevent an incontrollable outbreak of COVID-19. This is underlined by a Chinese study which reported that hospital-related transmission accounts for an estimated 41% of all hospitalized patients with COVID-19.^26^

If chest CT is employed as a screening tool, it should have (nearly) perfect sensitivity and NPV because only one missed COVID-19 can cause a disastrous contamination throughout the hospital. Specificity and PPV should be acceptably high, because even only one false negative COVID-19 case will result in unnecessary occupation of isolation rooms. However, sensitivity and NPV of frontline general radiologists were insufficiently high, whereas specificity and PPV were unacceptably low. Therefore, our results suggest that chest CT interpreted by frontline general radiologists cannot be used as a reliable, independent screening tool for COVID-19. Although specificity and PPV of a chest radiologist were higher, sensitivity and NPV were still insufficiently high.

Typical CT features of COVID-19 pneumonia reported in the recent literature include multifocal bilateral ground glass opacities with patchy consolidations, prominent peripherally subpleural distribution, and preferred posterior part or lower lobe predilection.^23^ False positive CT findings are encountered in patients with other viral pneumonias who have overlapping CT imaging features.^18^ The ongoing common flu season in our country during our study period,^27^ could have further limited the specificity of chest CT in our study. Interestingly, however, specificity of the chest radiologist was significantly higher than specificity of the frontline general radiologists (*p* = 0.001). Training and experience, but also the use of a predefined grading scale (CO-RADS)^24^ may be possible reasons why the chest radiologist achieved a higher specificity.

The findings of our study are in line with studies of Bernheim et al,^19^ Pan et al,^20^ and Yang et al,^21^ who also found a considerable number of false negative chest CT findings in COVID-19 patients. Accordingly, a recent meta-analysis showed that 10.6% of patients with COVID-19 have normal chest CT findings.^28^ In particular, chest CT findings can be negative early in the course of the disease,^19,20^ which was also the case in our study. Although Ai et al.^18^reported that the sensitivity of chest CT for the diagnosis of COVID-19 was near perfect, this is probably an overestimation because they investigated hospitalized patients who, compared with outpatients, are more likely to have abnormal CT findings.^29^ Of interest, a recent systematic review confirmed that the true sensitivity for CT based on unbiased studies is limited.^30^

Artificial intelligence (AI) may improve the diagnostic accuracy of chest CT. A recently published study showed that AI achieved 90% sensitivity (95% CI: 83%, 94%) and 96% specificity (95% CI: 93%, 98%).^31^ However, this is still insufficient to exclude COVID-19. It is questionable whether further development of AI software will achieve (near) perfect diagnostic screening performance because there are COVID-19 patients who do not have lung abnormalities yet in the early course of the disease.^18,19^ Furthermore, there is a considerable number of symptomatic patients with upper respiratory tract infections who not develop pneumonia.^10,32^

Our study has some limitations. First, only 33.3% of patients with negative initial RT-PCR result were retested. Because of the limited availability of RT-PCR kits in our hospital, it was not feasible to retest all patients with negative initial RT-PCR result. However, according to our reference standard, all patients with persistent clinical suspicion were retested. Second, initial chest CT interpretation was performed by 15 different frontline general radiologists. However, the use of different frontline general radiologists reflects clinical practice during the current COVID-19 crisis (frontline general radiologists have alternating shifts). Third, the frontline general radiologists were not experienced in assessing chest CT in COVID-19 and there may be a learning curve. Furthermore, they were not instructed to use explicit threshold criteria. This may have influenced either sensitivity or specificity, depending on the possible implicit criteria used by the frontline general radiologists.

In conclusion, our study suggests that chest CT interpreted by frontline general radiologists achieves insufficient screening performance. Although specificity of a chest radiologist appears to be significantly higher than that of frontline general radiologists, sensitivity did not improve. A negative chest CT result does not exclude COVID-19.

## Notes

Please change into: Krdzalic J, de Jaegere TMH, Kwee RM, COVID-19 CT Investigators South-East Netherlands (CISEN) Study Group.

Please change into: Krdzalic J, de Jaegere TMH, Kwee RM, COVID-19 CT Investigators South-East Netherlands (CISEN) Study Group
